# Ultrasonographic and Thermographic Evaluation in Brachycephalic Dogs Before and After Physical Exercise in the Brazilian Northeast Semi-Arid Region

**DOI:** 10.3390/vetsci13090906

**Published:** 2026-09-04

**Authors:** Amanda Carneiro Cardoso, Carina Rodrigues da Silva, Whítara Ferreira Lima, Ellen Medeiros Almeida, Ana Amélia Domingues Gomes, Rafael Felipe da Costa Vieira, Alexandre Redson Soares da Silva

**Affiliations:** 1 Graduate Program in Veterinary Sciences in the Semi-Arid Region, Agricultural Sciences Campus, Federal University of São Francisco Valley, Petrolina 56304-917, PE, Brazil; amanda.cardoso@discente.univasf.edu.br (A.C.C.); dracarina.cardiovet@gmail.com (C.R.d.S.); whitara.flima@discente.univasf.edu.br (W.F.L.); ellen.medeiros@discente.univasf.edu.br (E.M.A.); anaamelia.gomes@univasf.edu.br (A.A.D.G.); 2 Center for Computational Intelligence to Predict Health and Environmental Risks, University of North Carolina at Charlotte, Charlotte, NC 28223, USA; rvieira@charlotte.edu; 3 Department of Epidemiology and Community Health, University of North Carolina at Charlotte, Charlotte, NC 28223, USA

**Keywords:** thoracic ultrasound, hyperthermia, brachycephalic syndrome, thermal stress, B-lines, infrared thermography, exercise intolerance

## Abstract

Brachycephalic dogs are predisposed to respiratory and thermoregulatory impairment, which may become evident during physical exertion. This study evaluated thermal and pulmonary ultrasonographic responses to standardized submaximal treadmill exercise in 41 clinically healthy brachycephalic dogs. Rectal temperature and infrared thermographic measurements of the ocular and facial regions were obtained before and immediately after exercise. Thoracic ultrasonography was performed at baseline and after a standardized 20-min post-exercise recovery period. The dogs walked on a treadmill at 1.3 m/s, with a 0° incline, for up to 6 min. Rectal temperature increased significantly after exercise (*p* < 0.0001), and significant changes were also observed in ocular thermographic regions R1 and R2 (*p* < 0.0001), whereas facial surface temperature did not differ significantly. No pulmonary ultrasonographic abnormalities were detected at baseline; after exercise, abnormalities were identified in 30/41 dogs (73%), including B-lines, pleural irregularity, pleural thickening, and, less frequently, discrete pleural effusion. These findings demonstrate measurable thermal responses and pleuropulmonary ultrasonographic changes following standardized submaximal exercise in brachycephalic dogs. Infrared ocular thermography and thoracic ultrasonography may provide complementary, non-invasive information for monitoring exercise-associated responses; however, the relationship between ocular and core body temperatures and the temporal and clinical significance of the pulmonary findings require further investigation.

## Simple Summary

Brachycephalic dogs may experience respiratory and thermoregulatory challenges during physical activity because of their upper airway conformation. This study evaluated thermal and pulmonary ultrasonographic responses to standardized submaximal treadmill exercise in 41 clinically healthy brachycephalic dogs. Rectal temperature and infrared thermographic measurements were obtained before and immediately after exercise, whereas thoracic ultrasonography was performed before exercise and after a standardized 20-min recovery period. Exercise was followed by significant increases in rectal and ocular temperatures, while pulmonary ultrasonography identified post-exercise B-lines and pleural abnormalities that were absent at baseline. These findings indicate measurable thermal responses and pleuropulmonary ultrasonographic changes following exercise in the dogs evaluated. Infrared thermography and lung ultrasound may provide complementary, non-invasive information for monitoring exercise-associated responses in brachycephalic dogs; however, further controlled and serial studies are required to determine the clinical significance and temporal behavior of these findings.

## 1. Introduction

Brachycephalic syndrome, or Brachycephalic Obstructive Airway Syndrome (BOAS), results from anatomical abnormalities that cause upper airway obstruction, including stenotic nares, elongated soft palate, everted laryngeal saccules, laryngeal collapse, and, in some individuals, tracheal hypoplasia and laryngeal paralysis [1]. These malformations cause turbulent airflow through the nostrils, inspiratory stertor, soft tissue edema, and severe inspiratory dyspnea, which may progress to death. Pronounced respiratory effort may also be associated with gastrointestinal disorders, such as regurgitation or vomiting, resulting from excessive negative intrathoracic pressure [2]. The most frequently affected breeds include the French and English Bulldogs, Shih Tzu, Pug, Boxer, Pekingese, Boston Terrier, Shar Pei, Cavalier King Charles Spaniel, and Lhasa Apso [3].

Brachycephalic animals are predisposed to hyperthermia due to impaired peripheral thermoregulation secondary to anatomical malformations that compromise efficient heat dissipation [4]. Heatstroke is an acute condition influenced by external factors such as stress, high ambient temperature, and physical exercise, which can result in respiratory impairment and severe systemic alterations [5]. This vulnerability may be exacerbated in semi-arid environments, where high temperatures challenge the thermal homeostasis of these patients.

In this context, infrared thermography is a non-invasive tool that enables measurement of body surface temperature without requiring physical restraint [6]. Ocular temperature is a reliable indicator of core body temperature due to the anatomical proximity of the ocular region to the brain and immediate autonomic response [7,8]. Additionally, pulmonary ultrasonography is an accessible and safe diagnostic method for serial evaluations, as it does not require ionizing radiation [9]. This method is indicated for the screening and diagnosis of acute respiratory distress in brachycephalic dogs, allowing dynamic monitoring of therapeutic response [10,11,12].

Despite the recognition of the anatomical alterations that comprise BOAS, the early detection of parenchymal lung injuries resulting from thermal stress and mild physical exertion remains insufficiently investigated. It is hypothesized that respiratory effort in brachycephalic dogs triggers immediate structural lung alterations, detectable by imaging before evident clinical collapse. Therefore, the objective of this study was to evaluate, using pulmonary ultrasonography, the impact of low-intensity physical exercise on the lungs of brachycephalic dogs and to monitor the associated thermal response via ocular infrared thermography.

## 2. Materials and Methods

Experimental procedures were conducted following approval by the Institutional Ethics Committee on Animal Use (CEUA) of the Federal University of the São Francisco Valley (Univasf) under protocol no. 0001/260421.

### 2.1. Location and Animal Selection

The study was carried out during March and April 2022 in the Caatinga biome, located in the Brazilian Northeast. The climate is predominantly hot semi-arid, characterized by short rainy seasons of 3 to 5 months, typically from January to May, while the dry season spans 7 to 9 months for most of the year. The region features strong, dry winds, with an average annual temperature between 25 °C and 30 °C and relative humidity close to 50%. During the study period, mean temperature and humidity were 30.98 °C and 43.53% in March and 32.58 °C and 37.54% in April, respectively.

The study was conducted in partnership with a veterinary clinic located in Petrolina, Pernambuco, Brazil. A total of 41 brachycephalic dogs of both sexes, aged between 1 and 7 years, were included in the study. The inclusion criteria were: (1) brachycephalic conformation; (2) age between 1 and 7 years; (3) absence of clinically apparent systemic or cardiopulmonary abnormalities on physical examination; and (4) absence of detectable pre-existing cardiopulmonary abnormalities on thoracic radiographic screening. Thoracic radiography was performed using four standard projections (right and left lateral, ventrodorsal, and dorsoventral) before the experimental protocol. Dogs were excluded if physical examination or thoracic radiography revealed evidence of pre-existing cardiopulmonary disease or if their clinical condition precluded safe participation in the standardized treadmill exercise protocol. The final study population comprised French Bulldogs (*n* = 23), Pugs (*n* = 12), and Shih Tzus (*n* = 6).

### 2.2. Performance of Thermal, Sonographic, and Thermographic Assessments

Two distinct environments were utilized for the experiment: a naturally ventilated room for treadmill exercise and a climate-controlled room (22 °C) for imaging assessments. After radiographic screening, the animals remained in the climate-controlled environment for 20 min to allow clinical respiratory stabilization following the stress caused by physical restraint during the examination. Subsequently, the pre-exercise phase was initiated, consisting of rectal temperature measurement, capture of infrared thermographic images (Flir^®^ E6-XT, FLIR Systems, Inc., Wilsonville, OR, USA) of the ocular regions (R1: central region and R2: lacrimal limbus) and the superficial face, as well as baseline pulmonary ultrasound (Mindray Z5 Vet^®^, Shenzhen Mindray Bio-Medical Electronics Co., Ltd., Shenzhen, China).

Pulmonary ultrasonography was conducted using the regionally based Veterinary Bedside Lung Ultrasound Examination (VetBLUE) approach described by Lisciandro et al. (2014) [12]. Four standardized transthoracic acoustic windows were systematically evaluated in each hemithorax, comprising the caudodorsal (Cd), perihilar (Ph), middle (Md), and cranial (Cr) lung regions, for a total of eight pulmonary scanning sites. The pleural–pulmonary interface was assessed at each site for the presence and distribution of B-lines and for pleural abnormalities, including pleural irregularity, pleural thickening, and pleural effusion. The same scanning protocol was applied during the pre- and post-exercise examinations, and all examinations were performed by the same experienced operator to minimize interobserver variability [12]. B-lines were identified as discrete, hyperechoic vertical reverberation artifacts arising from the pleural–pulmonary interface and extending to the far field of the ultrasound image. Their presence and distribution across the standardized VetBLUE regions were recorded.

The physical exertion protocol was performed after adaptation to the treadmill at room temperature to simulate daily activity. During the procedure, the dogs wore a safety harness and received positive reinforcement with treats. The equipment was configured at a 0° incline and a speed of 1.3 m/s, maintaining a walking pace for a maximum period of 6 min. The treadmill speed of 1.3 m/s was selected because it represented the lowest speed available on the equipment that allowed the dogs to maintain a comfortable and continuous walking gait. The protocol was designed to provide a standardized, brief, submaximal walking stimulus rather than an individualized workload based on each dog’s maximal exercise capacity. This speed is within the range of submaximal treadmill speeds previously reported for standardized exercise testing in brachycephalic dogs [13,14]. The maximum 6-min duration was selected based on the conceptual framework of the 6-min walk test commonly used for functional assessment, while predefined stopping criteria allowed earlier termination according to individual exercise tolerance. Because a fixed treadmill speed may represent different relative workloads according to breed, body size, gait characteristics, and BOAS severity, the exercise stimulus should be interpreted as standardized rather than physiologically equivalent across all dogs.

**Figure 1:**
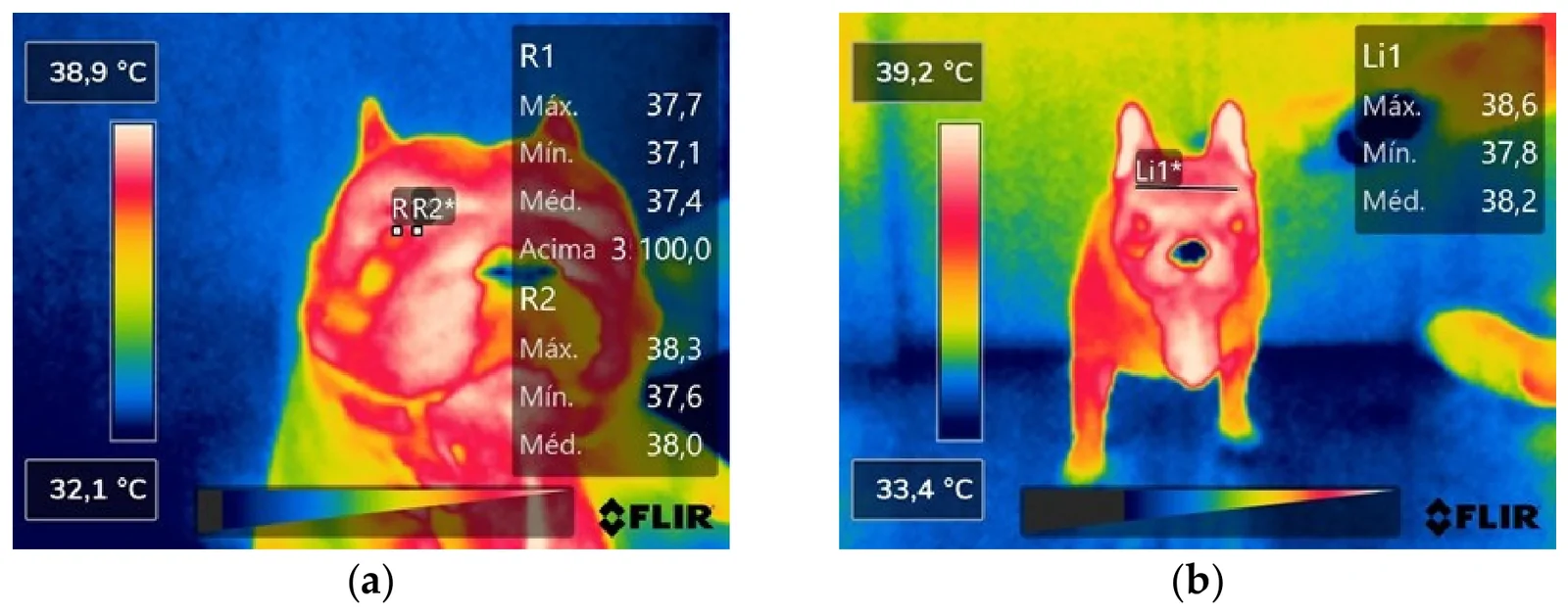
(**a**) Maximum and minimum temperatures obtained from the analysis of post-exercise ocular thermographic images. (**b**) Maximum and minimum temperatures obtained from the analysis of the superficial face. The asterisks (*) displayed next to the ROI labels are automatically generated by the FLIR thermographic analysis software and do not indicate statistical significance.

Throughout the test, respiratory patterns, open-mouth breathing, and the presence of nasal discharge were rigorously monitored. If the animal exhibited severe tachypnea, extreme fatigue, or excessive nasal discharge, the activity was immediately interrupted for safety reasons. Immediately following the walk, the animals underwent a new rectal temperature measurement and capture of ocular and facial thermographic images (Figure 1).

**Figure 2:**
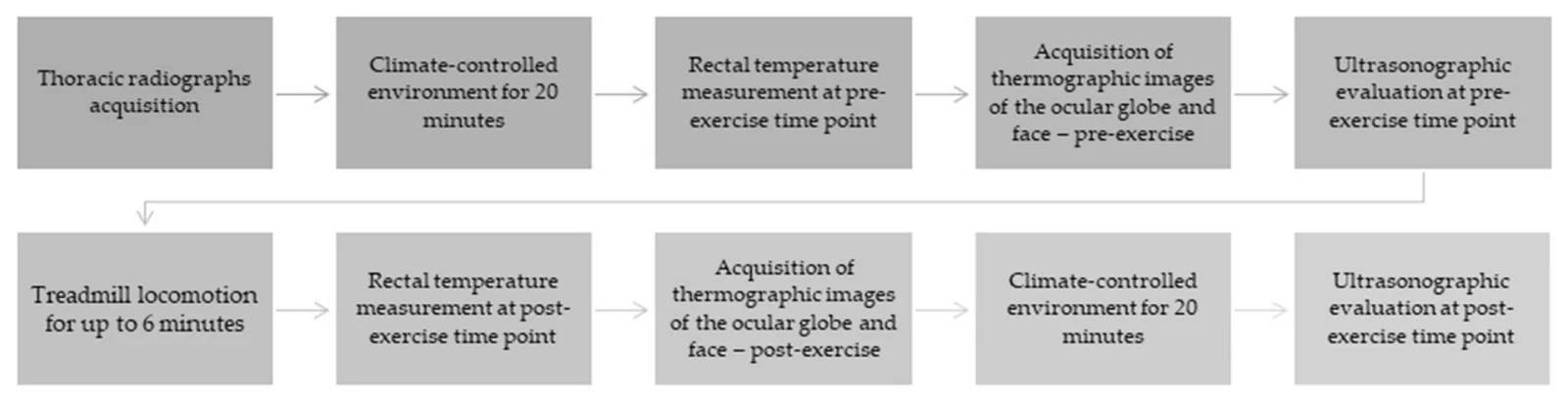
Timeline of the study assessment methodology. Arrows indicate the chronological sequence in which the procedures and assessments were performed.

Following thoracic radiographic screening using four standard projections (right and left lateral, ventrodorsal, and dorsoventral), the dogs were returned to a temperature- and humidity-controlled room for a standardized 20-min recovery period to allow attenuation of marked post-exercise tachypnea and facilitate standardized ultrasound acquisition. Pre-exercise evaluations included rectal temperature measurement, infrared thermographic imaging of the ocular and facial regions, and thoracic ultrasonography. The dogs then underwent treadmill walking for a maximum of 6 min at a 0° incline and a speed of 1.3 m/s. Immediately after exercise, rectal temperature and infrared thermographic measurements were repeated to capture the acute thermal response. The dogs were then returned to the temperature- and humidity-controlled room for a standardized 20-min recovery period before the post-exercise thoracic ultrasound examination. This recovery interval was incorporated to prioritize animal welfare, allow attenuation of marked post-exercise tachypnea, and facilitate standardized ultrasound acquisition with reduced respiratory motion. Accordingly, thermal measurements characterized the immediate post-exercise response, whereas pulmonary ultrasonography characterized pleuropulmonary findings detectable after the standardized early recovery period. A detailed flowchart of the experimental sequence is presented in Figure 2.

### 2.3. Statistical Analysis

Quantitative data were expressed as mean, standard deviation (SD), and standard error of the mean (SEM). The normal distribution of the variables was evaluated using the D’Agostino–Pearson test. For comparison of rectal temperature between pre- and post-exercise time points, the paired Student’s *t*-test was utilized, as the data followed a normal distribution. Infrared thermography data regarding the maximum temperatures of the ocular regions (R1 and R2) and the superficial face were analyzed using the Friedman test due to the absence of normality. When a significant effect was identified, Dunn’s post hoc test was applied for multiple comparisons. To evaluate the interaction between the temperatures in the different regions (ocular and facial) and the time points (pre- and post-exercise), a two-way repeated-measures analysis of variance (Two-way RM ANOVA) was employed. In this analysis, time point and the evaluated regions were considered as factors, followed by Sidak’s post-hoc test for multiple comparisons, where appropriate. Pulmonary ultrasound findings were analyzed using descriptive statistics and expressed as percentages. The significance level adopted for all analyses was 5% (*p* < 0.05). All statistical procedures were performed using GraphPad Prism version 7.0. Thermographic images were processed using FLIR Thermal Studio software, version 1.9.10, to obtain maximum and minimum temperatures.

## 3. Results

### 3.1. Clinical and Behavioral Parameters

Comparison of the means of initial and final rectal temperatures revealed a significant increase after the treadmill exercise (*p* < 0.0001), with a mean difference of 0.3512 °C, highlighting the influence of physical exertion on the evaluated animals. The average duration of the exercise was up to 6 min.

Regarding the behavior of the animals subjected to treadmill exertion, only 5% showed no changes. It was observed that 46% of the dogs began or exhibited open-mouth breathing during the exercise; 22% showed open-mouth breathing associated with mild nasal discharge; 2% opened their mouths after 5 min of exercise; 2% exhibited open-mouth breathing with mild nasal discharge and salivation; 10% manifested open-mouth breathing and moderate nasal discharge; 2% exhibited only moderate nasal discharge; 7% did not complete the full exercise period on the treadmill; and 2% did not complete the full exercise period and presented open-mouth breathing with moderate nasal discharge.

### 3.2. Thermographic Evaluation

**Figure 3:**
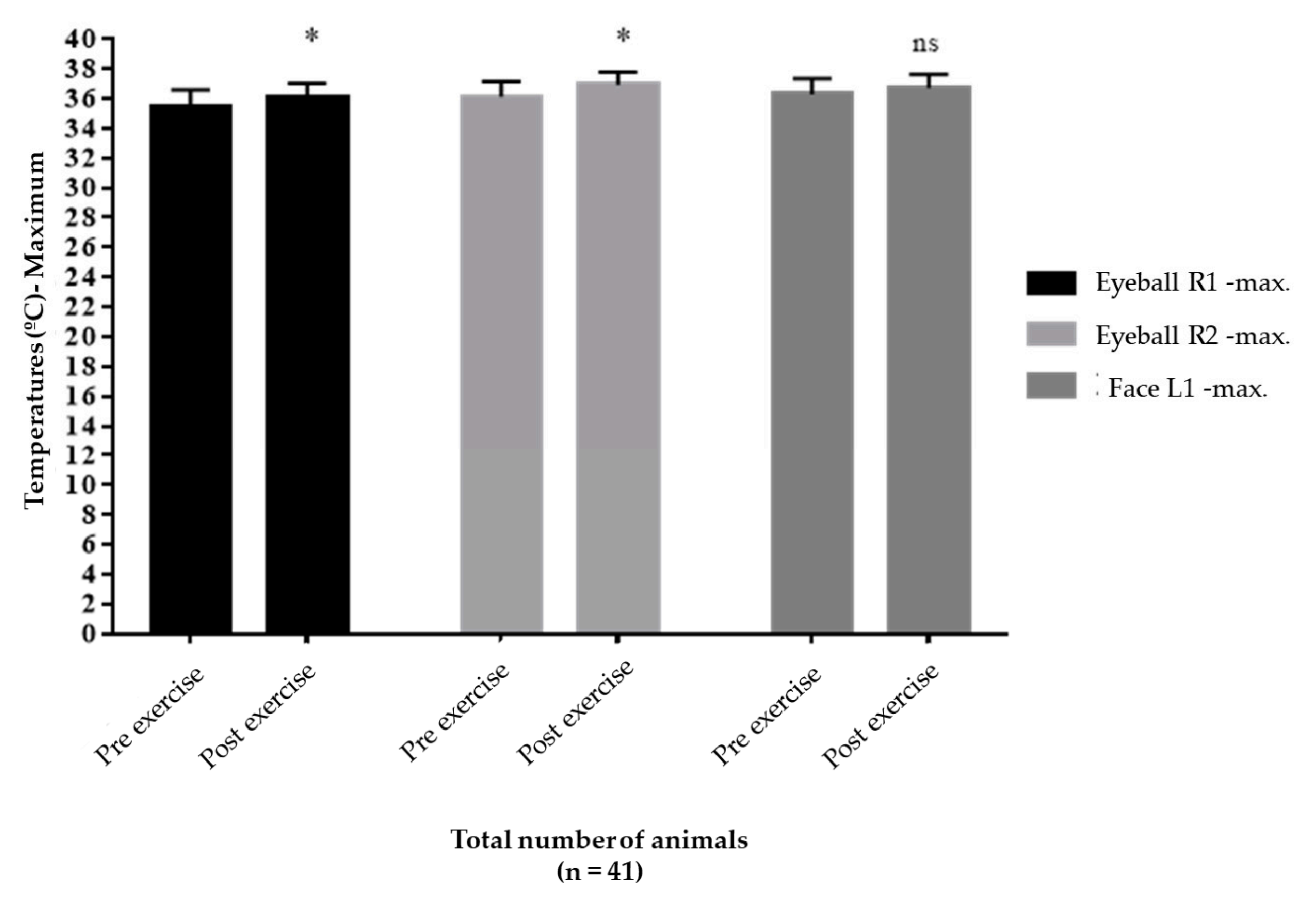
Comparison of maximum eyeball temperatures obtained pre- and post-exercise using infrared thermography. * indicates a statistically significant difference (*p* < 0.05); ns indicates a non-significant difference (*p* > 0.05).

In the thermographic evaluation, maximum temperatures were compared for the eyeball region (R1: central region and R2: lacrimal limbus) and the superficial face under pre- and post-exercise conditions. The overall comparison was significant (*p* < 0.0001; Friedman statistic = 78.74). Dunn’s post-hoc test indicated that significant differences occurred only in the eyeball regions. In the R1 region, the adjusted *p*-value was 0.0151, while in R2, the adjusted *p*-value was 0.0019. The mean facial thermographic evaluation did not differ significantly, with an adjusted *p*-value of 0.0806 (Figure 3).

**Figure 4:**
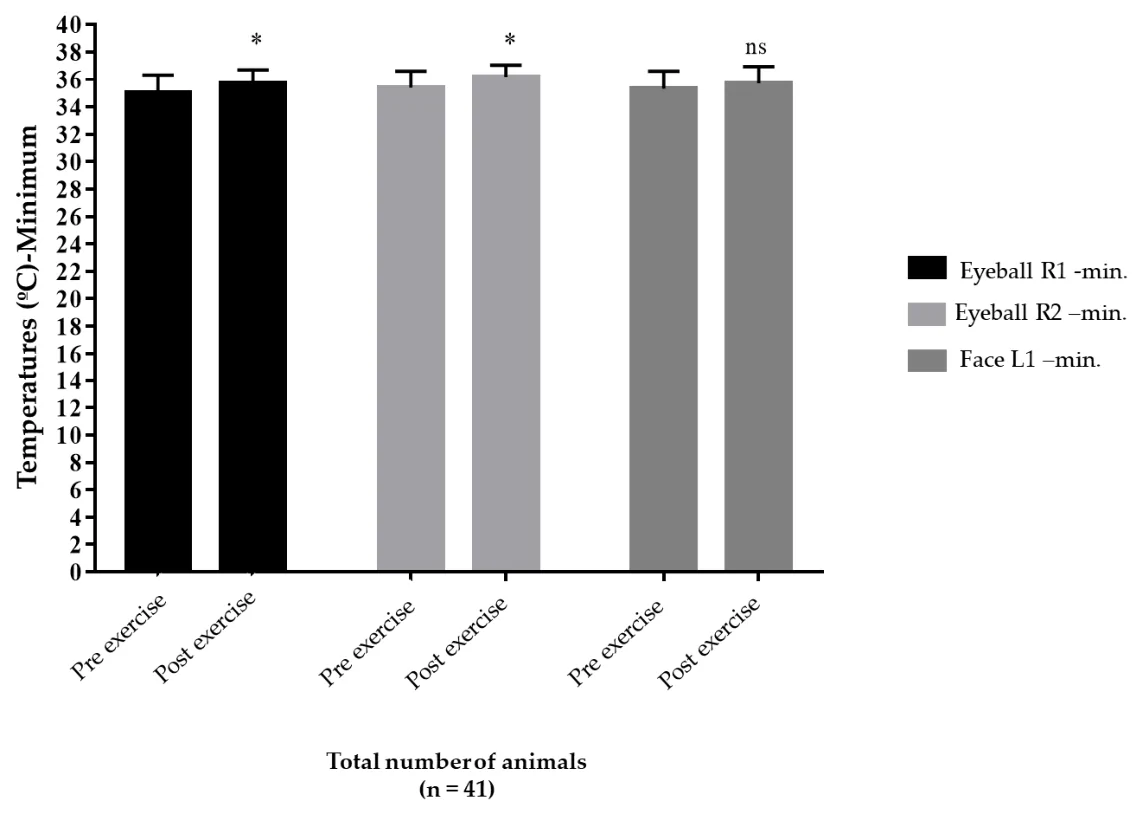
Comparison of minimum eyeball temperatures obtained pre- and post-exercise using infrared thermography. * indicates a statistically significant difference (*p* < 0.05); ns indicates a non-significant difference (*p* > 0.05).

Regarding the minimum temperature, the data also showed a significant difference (*p* < 0.0001; Friedman statistic = 55.2). Again, only the thermographic measurements related to the pre- and post-exercise comparison of the eyeball regions (R1 and R2) were significant, while the mean facial values exhibited no differences. The adjusted *p*-values for R1 and R2 were 0.0043 and 0.0008, respectively. The facial values were not significantly different, with an adjusted *p*-value of 0.6787 (Figure 4).

Two-way RM ANOVA was applied to evaluate the eyeball and face groups. Significant differences were found only for the period factor (*p* = 0.443). The other sources showed no differences, with *p* = 0.9941 for the interaction and *p* = 0.9968 for temperature. Multiple comparisons using Sidak’s test indicated no significant differences between groups.

### 3.3. Lung Ultrasound Evaluation

Regarding lung ultrasound findings under pre-exercise conditions, none of the 41 animals showed any changes. In the post-exercise period, 73% (*n* = 30) exhibited sonographic changes, while 27% (*n* = 11) did not manifest any alterations (Table 1).

**Figure 5:**
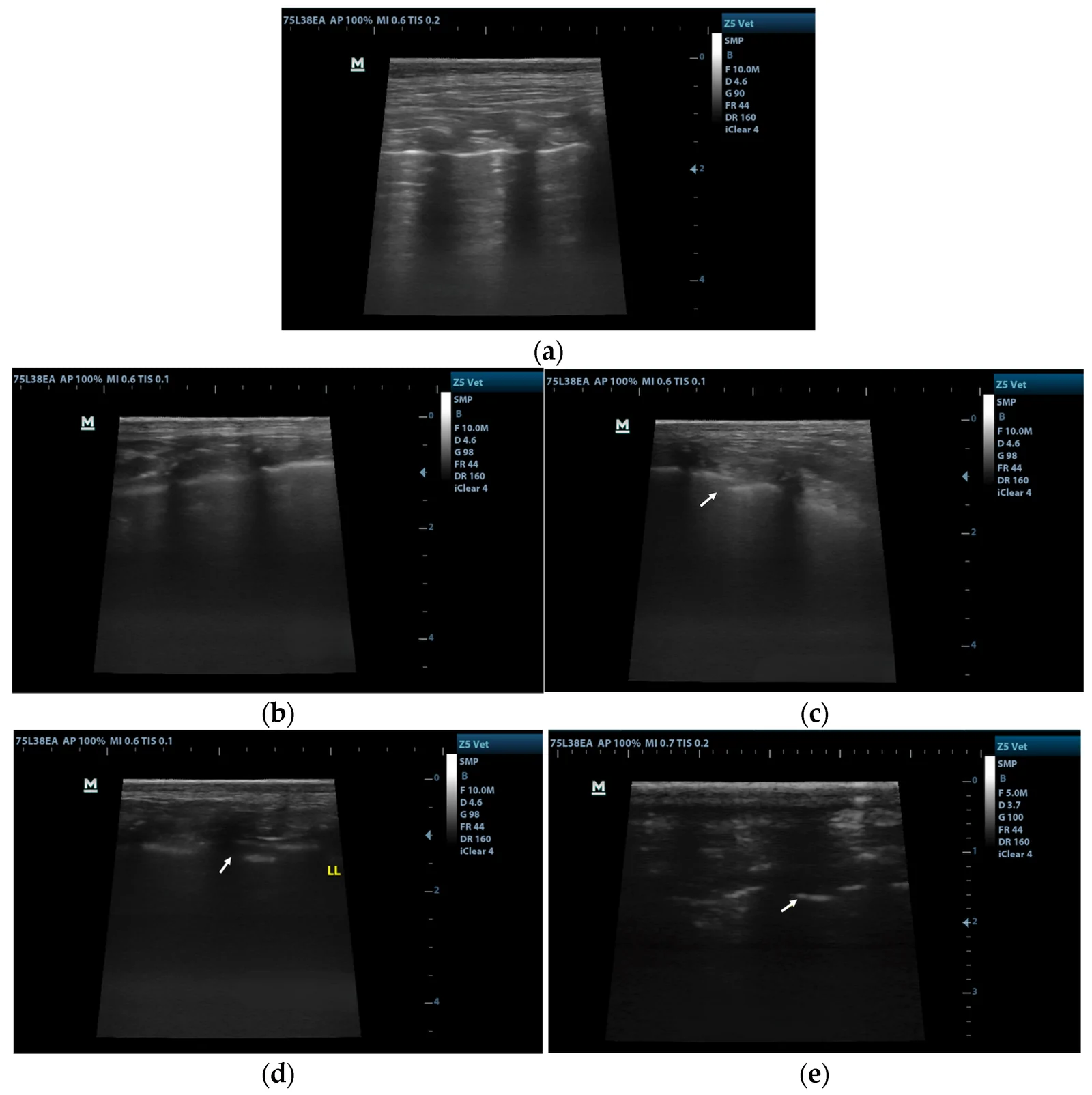
Sonographic images highlighting the main alterations. (**a**) Sonogram of a dog during pre-exercise evaluation, showing a normal lung. (**b**,**c**) Images showing pleural irregularity/thickening and B-lines. (**d**,**e**) Images of pleural effusion (white arrow).

Among the animals that exhibited changes (*n* = 30), 50% (*n* = 15) presented pleural thickening. Regarding pleural irregularity, 70% (*n* = 21) of the dogs exhibited this alteration, while the remaining 30% (*n* = 9) did not. Regarding pleural effusion, 90% (*n* = 27) of the evaluated dogs did not exhibit fluid accumulation in the pleural space; the 10% (*n* = 3) of animals that did present the alteration exhibited effusion in the regions of the middle lobe, peri-hilar lung lobe, and cranial lobe. Concerning B-lines, it was observed that 72% (*n* = 21) of the animals presented this alteration during the ultrasound examination (Figure 5).

## 4. Discussion

In dogs, heat dissipation occurs predominantly through evaporative cooling associated with panting, as the species has a limited number of functional sweat glands [15]. In brachycephalic dogs, anatomical abnormalities of the upper airways increase airflow resistance and significantly elevate respiratory effort, reducing thermoregulation efficiency and increasing susceptibility to hyperthermia, especially during exercise or thermal stress [16,17].

The 6-min walk test has been widely used in veterinary medicine, particularly in studies focused on cardiovascular assessment. Its application was initially described by Boddy et al. in 2004 [17] and subsequently employed by Cerda-Gonzalez et al. in 2016 [18]. In the present study, the test was adapted to evaluate exercise tolerance in brachycephalic dogs and to investigate potential respiratory repercussions associated with physical exertion.

In this study, the animals were subjected to light physical exercise, simulating a walk with the owner, and were not exposed to fatiguing activity. Consequently, no dog exhibited signs of heatstroke during the experiment. Because the study was not designed to stratify dogs according to BOAS severity, the observed physiological responses should be interpreted as representative of a clinically heterogeneous brachycephalic population rather than as specific to any degree of upper airway obstruction. Even under low-intensity physical and/or thermal stimuli, brachycephalic dogs showed temperature changes post-exercise. These findings indicate that even under low-intensity or submaximal exercise conditions, brachycephalic dogs demonstrate measurable post-exercise thermal responses. Considering that body temperature reflects the dynamic balance between metabolic heat production and the rate of thermal dissipation, assessments were performed at two distinct moments: pre-exercise and post-exercise.

In the study by Davis et al. [16], brachycephalic dogs, when compared with non-brachycephalic animals subjected to the same thermal stress, exhibit significantly higher respiratory rates and body temperatures. These findings reinforce that brachycephalic dogs have a lower capacity to maintain thermoregulation under stressful conditions compared with mesocephalic or dolichocephalic animals.

Respiratory manifestations are the most frequent clinical signs in dogs affected by brachycephalic syndrome [19]. Obstruction of the upper airways, particularly due to stenotic nares, promotes increased airflow resistance, with inspiratory dyspnea being the clinical sign of this obstructive pattern [20]. As the syndrome progresses, the severity of the respiratory condition may increase, particularly when associated with secondary changes in the larynx and trachea, further compromising ventilatory efficiency and thermoregulatory capacity [5,21,22].

The variation in ocular temperature may be associated with activation of the sympathetic component of the autonomic nervous system, representing one of the initial phases of the stress response [23]. In the presence of a stressful stimulus, the hypothalamic–pituitary–adrenal axis is activated, resulting in increased cortisol release and intensified metabolism, which can influence both body heat production and dissipation [24]. Accordingly, the ocular thermal increase detected herein suggests that even light exercise may trigger an immediate protective autonomic response in these animals.

In the study by Pulido-Rodríguez et al. (2017) [25], infrared ocular thermography was used in nursery pigs to assess stress. Two distinct regions were analyzed: the lacrimal caruncle and the posterior region of the eyeball. The authors observed significantly higher temperatures in the region near the lacrimal caruncle than in the posterior region. Although the study was conducted in a different species, these results are similar to those found in the present study of brachycephalic dogs.

Ocular temperature may be related to core temperature due to the anatomical proximity of the ocular region to the brain. However, although both rectal and ocular temperatures increased following exercise in the present study, no individual-level correlation or agreement analysis was performed between these measurements. Therefore, our findings should not be interpreted as demonstrating that ocular temperature is an accurate surrogate for core body temperature. For this reason, the eye and retroauricular region are considered reliable points for the indirect assessment of surface temperature via infrared thermography [24,26]. Studies in cattle [23] and horses [8,27] have also demonstrated that areas of the ocular surface near the lacrimal limbus exhibit higher temperatures than other regions of the eyeball.

There is still no consensus on why the lacrimal limbus region exhibits elevated temperatures. However, this finding is believed to be related to higher local blood flow and the presence of larger-caliber vessels in the medial conjunctiva compared with those in the central region of the eye [28]. Furthermore, the lack of significant variation in the facial surface, in contrast to the eyeball, reinforces the potential selectivity of the ocular region as an early indicator of thermal stress, possibly due to less interference from factors such as thermal insulation provided by the facial skin folds typical of these breeds.

Considering that the increase in body temperature in brachycephalic dogs may be associated with both increased respiratory effort and anatomical limitations of the upper airways, it is relevant to investigate whether such functional changes could be accompanied by detectable modifications in the lung parenchyma. In this context, lung ultrasound was employed in the present study to evaluate potential pulmonary changes associated with light physical exercise.

Notably, none of the dogs exhibited pulmonary ultrasonographic abnormalities at baseline, whereas post-exercise changes were detected in 73% (30/41) of the animals. Because the post-exercise lung ultrasound examination was performed after a standardized 20-min recovery period, the detection of ultrasonographic abnormalities suggests that these changes were not restricted to the immediate exercise phase. However, because no serial ultrasonographic examinations were performed beyond this time point, their duration and reversibility could not be determined. Therefore, these findings may represent transient exercise-associated physiological or subclinical pulmonary responses, although their potential pathological significance cannot be established from the present data.

In the acute inflammatory process of the pleura, termed pleuritis, thickening and irregularity of the pleural line may occur, with the extent varying according to the severity of the inflammatory process. Pleural thickening makes the sliding surfaces rougher and more irregular, potentially compromising respiratory dynamics, especially when the visceral pleura is involved. Ultrasonographically, this thickening may appear smooth or be associated with irregularities at the pleural interface [29].

Discrete pleural effusion was detected in 10% (3/30) of dogs with post-exercise ultrasonographic abnormalities. Because no fluid sampling or serial ultrasonographic follow-up was performed, its composition, underlying mechanism, and duration could not be determined. Pleural effusion can be triggered by various pathological conditions, including inflammatory, infectious, and neoplastic processes related to both the pleura and other structures of the thoracic cavity. Ultrasound is a sensitive method for confirming pleural effusion and can provide information regarding its content characteristics and estimated accumulated volume [29].

Pulmonary involvement with fluid accumulation in the interstitium generates ultrasonographic artifacts originating at the pleural surface, previously described as “comet tails” and currently termed B-lines. These are produced when small alveoli, which fall below the resolution limit of the sound beam, are filled with fluid while remaining surrounded by air. Ultrasonographically, they appear as vertical, non-attenuating hyperechoic lines extending from the pleuropulmonary interface to the bottom of the image. Isolated visualization of one or two discrete B-lines per field has no clinicopathological significance [29].

B-lines constitute the main sonographic finding of interstitial lung diseases. In these conditions, partial replacement of alveolar air content by a small amount of liquid occurs, which may represent an initial phase of interstitial pulmonary edema [30,31]. B-lines were among the most frequent post-exercise findings. Although an increased number of B-lines may occur in conditions associated with reduced peripheral lung aeration or increased interstitial fluid, this artifact is not specific for pulmonary edema and should not, in isolation, be interpreted as evidence of disruption of the blood–air barrier. In the present study, their absence at baseline and detection after exercise indicate an exercise-associated change; however, the underlying mechanism remains uncertain.

In brachycephalic dogs, increased upper airway resistance can generate greater negative intrathoracic pressure during inspiration, providing a biologically plausible mechanism by which marked respiratory effort could influence pulmonary hemodynamics. This change may result in pulmonary hemodynamic modifications, including elevation of capillary hydrostatic pressure and consequent extravasation of fluid into the pulmonary interstitium (non-cardiogenic acute pulmonary edema). This condition has been described as a potential complication of thermal stress or physical exercise and can progress to severe states and is considered a cause associated with sudden death in these animals [32]. Thus, sympathetic activation and the increase in ocular temperature may represent indicators of physiological stress preceding these hemodynamic changes in the lung parenchyma.

### Strengths and Limitations

Despite the methodological rigor, the present study has limitations that must be considered. First, clinical grading of the severity of BOAS was not performed for each animal, which precluded correlation between the degree of anatomical obstruction and the magnitude of the observed changes. Another limitation of this study is the absence of a non-brachycephalic control group. This study was intentionally designed as a paired before-and-after investigation, in which each dog served as its own physiological control. This approach minimized inter-individual variability and allowed direct assessment of exercise-induced physiological changes within the same animal. However, this design does not allow us to determine whether the observed thermographic and pulmonary ultrasonographic responses are exclusive to brachycephalic dogs or represent a general physiological response to exercise. Therefore, comparative studies including mesocephalic and dolichocephalic dogs are warranted.

Post-exercise rectal temperature and infrared thermographic measurements were obtained immediately after treadmill exercise, whereas thoracic ultrasonography was performed following a standardized 20-min recovery period. Therefore, these assessments represent different temporal components of the post-exercise response and should not be interpreted as directly synchronized measurements. Although the standardized recovery period applied to the ultrasound assessments improved methodological consistency and animal welfare, the study design does not allow determination of the exact onset or peak of the pulmonary ultrasonographic changes or their direct temporal relationship with the immediate thermal response. Serial studies integrating thermography and lung ultrasound at matched post-exercise time points are warranted to characterize these temporal relationships.

Although significant post-exercise changes were observed in both rectal and ocular temperatures, the relationship between these measurements was not formally evaluated using correlation, regression, or agreement analyses. Consequently, the present study cannot establish ocular thermography as a reliable surrogate for core body temperature or determine its diagnostic accuracy. Future method-comparison studies incorporating Bland–Altman analysis and regression approaches are required to determine the agreement and predictive relationship between ocular and rectal temperatures. Diagnostic performance measures, including sensitivity and specificity, would additionally require predefined clinically relevant temperature thresholds.

Overall, the findings of the present study demonstrate that even under light physical exercise, brachycephalic dogs exhibit thermal changes detectable through ocular thermography, accompanied by discrete pulmonary ultrasonographic changes compatible with interstitial and pleural involvement. These results suggest that anatomical limitations of the upper airways can trigger measurable, although subclinical, physiological responses to low-intensity stimuli. The association between increased body temperature, potential autonomic activation, and pulmonary changes suggests that these animals may have lower respiratory functional reserve and greater susceptibility to thermal imbalances, even under controlled conditions. Thus, the combined use of infrared thermography and lung ultrasound may be a promising complementary approach for the early assessment of respiratory repercussions in brachycephalic dogs subjected to physical exertion.

## 5. Conclusions

Standardized submaximal treadmill exercise was followed by significant increases in rectal and ocular temperatures and by detectable pleuropulmonary ultrasonographic changes in the brachycephalic dogs evaluated. Infrared ocular thermography provided a non-invasive approach for assessing exercise-associated changes in ocular surface temperature; however, because agreement and predictive performance relative to rectal temperature were not evaluated, it should not be interpreted as a validated surrogate for core body temperature. Thoracic ultrasonography identified B-lines and pleural abnormalities that remained detectable after a standardized 20-min recovery period in dogs with no ultrasonographic abnormalities at baseline. Therefore, infrared ocular thermography and thoracic ultrasonography may provide complementary, non-invasive information for monitoring thermal and pulmonary responses to exercise in brachycephalic dogs. Further controlled studies incorporating non-brachycephalic comparison groups, BOAS severity grading, and synchronized serial assessments are required to determine the phenotype specificity, temporal dynamics, and clinical relevance of these findings.

## Figures and Tables

**Table 1:** Absolute (*n*) and relative (%) frequency distribution of pulmonary ultrasound alterations and quantification of B-lines in the post-exercise period (*n* = 41).

Variables Analyzed	Absolute Frequency (*n*)	Relative Frequency (%)
General Ultrasound Findings		
Presence of changes	30	73.17%
Absence of changes	11	26.83%
Type of Change (*n* = 30)		
B-lines	21	72.00%
Pleural irregularity	21	70.00%
Pleural thickening	15	50.00%
Pleural effusion	3	10.00%
Quantification of Windows with B-Lines (*n* = 21)		
1 to 3 windows	10	47.62%
3 to 6 windows	8	38.10%
6 to 8 windows	3	14.28%

Percentages were calculated based on the number of animals that were exhibited in each condition (*n* = 30 for alterations; *n* = 21 for windows). Missing data corresponds to animals without post-exercise alterations (*n* = 11). The average treadmill exercise time was up to 6 min.

## Data Availability

The original contributions presented in this study are included in the article. Further inquiries can be directed to the corresponding author.
